# An Improved Method of Parameter Identification and Damage Detection in Beam Structures under Flexural Vibration Using Wavelet Multi-Resolution Analysis

**DOI:** 10.3390/s150922750

**Published:** 2015-09-09

**Authors:** Seyed Alireza Ravanfar, Hashim Abdul Razak, Zubaidah Ismail, Hooman Monajemi

**Affiliations:** 1 StrucHMRS Group, Department of Civil Engineering, Faculty of Engineering, University of Malaya, 50603 Kuala Lumpur, Malaysia; E-Mails: r.ravanfar@gmail.com (S.A.R.); hooman_monajemi@yahoo.com (H.M.); 2 Department of Civil Engineering, Faculty of Engineering, University of Malaya, 50603 Kuala Lumpur, Malaysia; E-Mail: zu_ismail@um.edu.my

**Keywords:** beam structure under flexural vibration, relative wavelet packet entropy, damage detection, wavelet transform, time-varying system identification

## Abstract

This paper reports on a two-step approach for optimally determining the location and severity of damage in beam structures under flexural vibration. The first step focuses on damage location detection. This is done by defining the damage index called relative wavelet packet entropy (RWPE). The damage severities of the model in terms of loss of stiffness are assessed in the second step using the inverse solution of equations of motion of a structural system in the wavelet domain. For this purpose, the connection coefficient of the scaling function to convert the equations of motion in the time domain into the wavelet domain is applied. Subsequently, the dominant components based on the relative energies of the wavelet packet transform (WPT) components of the acceleration responses are defined. To obtain the best estimation of the stiffness parameters of the model, the least squares error minimization is used iteratively over the dominant components. Then, the severity of the damage is evaluated by comparing the stiffness parameters of the identified model before and after the occurrence of damage. The numerical and experimental results demonstrate that the proposed method is robust and effective for the determination of damage location and accurate estimation of the loss in stiffness due to damage.

## 1. Introduction

Damage detection and condition monitoring of structures have become ever-increasing concerns. Most of the available techniques are based on visual inspection or localized assessment procedures such as ultrasonic, acoustic and impact echo which can be generated due to characteristic changes in the material. The most significant challenge for these methods is that they usually provide qualitative insight of the condition of structures rather than quantitative information. In addition, in some of these methods the location of damage is a priori and the accessibility of damage location is required [1]. Thus, global monitoring techniques in general and vibration-based monitoring methods in particular have been introduced and developed as a consequence of these limitations.

Structural identification using vibration data is a feasible strategy that can be widely applied in structural health monitoring (SHM), structural control, and structural response prediction [2,3]. The purpose of structural health monitoring is to evaluate the condition of the structure and to identify damage when it occurs. For this reason, analysis techniques that identify structural damage using vibration data obtained from sensors have garnered widespread interest. A variety of approaches have been mentioned in the literature for system identification and damage detection [4,5,6,7]. The theory of vibration-based SHM is that the dynamic characteristics of a structure are a function of its physical properties. When there are changes in these physical properties, such as a decrease in stiffness due to localized structural damage, there will be corresponding changes in the dynamic characteristics of the structure.

The SHM system generates abundant amounts of data; therefore, data processing and result interpretation become an important and challenging problem. The system identification algorithms are utilized to process the data while the vibration theories are used to extract features of the structure.

A commonly adopted approach to the identification of a structural system is through modal analysis. Identification of the modal characteristics of a structure can be carried out either by time-domain or frequency-domain methods. When it comes to considering a system’s properties with respect to time, frequency domain methods are not so adaptable. On the other hand, time domain methods are usually sensitive to noise due to the presence of all the frequency components in the data. To overcome these difficulties, several techniques have been developed in recent years that utilize time-scale or time-frequency domain analysis. Many of these methods are based on wavelet analysis, with application to linear and non-linear systems, because of its ability to retain information of local frequency content and variation with time, together with the advantage of flexible windowing over short-time Fourier transform (STFT). The use of the wavelet transform is particularly advantageous in problems related to system identification. During the identification process, the property of multi-resolution analysis in discrete wavelet transform can filter out the measurement noise from the structural response without the use of additional filters. Furthermore, the wavelet transform coefficients can be associated directly with the structural parameters [8].

The wavelet-based identification approaches of system parameters can be divided into two groups. The researchers in the first group employed the continuous wavelet transform (CWT)-based approach, whereas those in the second group used the discrete wavelet transform (DWT)-based approach.

A class of identification method was developed by Ruzzene *et al.* [9] and Staszewski [10] based on the continuous wavelet transform (CWT) to identify the natural frequencies and damping ratios of a structural system. Lardies and Gouttebroze [11] developed a CWT approach to identify damping ratios of closely spaced mode systems, an approach that applied a modified Morlet wavelet function and demonstrated better resolution than the CWT approach developed by Staszewski. Besides the Morlet wavelet, the Cauchy wavelet was introduced to overcome the identification limitation of linear time-variant parameters by Argoul and Le [12]. Slavič *et al.* [13] employed a CWT approach based on the Gabor wavelet function to estimate damping ratios. An extension towards the application of the CWT to the identification of time-varying systems was suggested by Curadelli *et al.* [14], Le and Paultre [15], and Wang *et al.* [16], Nagarajaiah and Basu [17].

Another class of identification method utilized discrete wavelet transform (DWT). Robertson *et al.* [18] used discrete Daubechies (DB) wavelet transform to extract the impulse response functions from input and output data. Then, the damping parameters and mode shapes of a system were obtained using state-space algorithm. Ghanem and Romeo [19] proposed a discrete wavelet identification approach to analyze time-varying structures which was associated with a differential equation model that relates input and output responses using wavelet Galerkin approach. Huang *et al.* [20] applied the DWT to discrete equations of motion and determined the modal properties of the structure using either earthquake or free decay responses. The DWT for linear time-invariant of non-parametric system identification was further explained by Luk and Damper [21]. Wavelet multi-resolution approximation for identification of arbitrary time-varying parameters was proposed in a shear beam. The wavelet multi-resolution approximation can be applied to estimate the instantaneous time-varying damping and stiffness, associated with the restoring forces. A wavelet-based state-space method was developed by Xu *et al.* [22] to identify dynamic parameters in linear time-varying systems. The method does not require computation of the second connection coefficients, as compared with the linear time-varying identification method presented by Shen and Law [23].

The detection of damage in a structure through the use of the wavelet theory has been extensively investigated by several researchers [24,25,26,27,28,29,30,31,32,33,34,35,36,37]. In these studies, while the damaged structure was frequently a beam, other elements, such as plates and a bidimensional structure, were also considered. Douka *et al.* [38] applied four symmetrical wavelet transforms on the mode shape to identify cracks in plate structures. The sudden change in the wavelet coefficients determines the location of the crack and the intensity factor was defined to approximate the depth of the crack from the coefficients of the wavelet transform. A two-dimensional directional Gaussian wavelet transform was proposed by Xu *et al.* [39] on operating deflection shapes for damage detection in plates.

The wavelet entropy, which is a combination of entropy and wavelet, could take advantage of both methods to explain the characteristics of a signal, which are not directly visible in the original space. The wavelet entropy is modified to give a damage signature, which can both be achieved at different time stations and spatial locations to identify the existence of damage [40]. Lee *et al.* [41] proposed a new damage detection algorithm based on the continuous relative wavelet entropy (CRWE) for truss bridge structures. The damage-sensitive index (DSI) of each sensor’s location was defined by CRWE measurements of different sensor-to-sensor pairs. The CRWE was reported to be able to detect damage but with considerably large computation cost for the real-time monitoring algorithm. In particular, Ren and Sun [42] suggested a combination of information entropy [43] and discrete wavelet transform having a damage-sensitive feature to characterize the level of irregularity in the measured signals to detect the occurrence and location of damage in beam structures.

This paper presents the two-step method using RWPE and system identification based on wavelet multi-resolution analysis to optimally determine the location and severity of damage in beam structures under flexural vibration. In the first step, the damage location is investigated by the new approach based on the combination of the WPT and entropy analysis. Accordingly, a new effective damage index (RWPE) is introduced to obtain information on the relative energy correlated with various frequency bands existing in structural response segments. In the second step, the system identification technique is employed to identify the stiffness parameter based on the inverse solution of the equation of motion of a structural system in the time-scale domain. The connection coefficients for the scaling function are developed to derive the velocity and displacement from the measured acceleration responses. In order to reduce the data size without losing any important features of the system, the dominant components based on the relative energy of WPT components are defined. To evaluate the severity of damage in terms of the loss of stiffness in the model, the least squares error minimization is employed repeatedly over the dominant components to achieve the best estimation of the parameters before and after the occurrence of damage. The simulation of a fixed-support beam with multiple damages is presented to test the proposed method.

## 2. Wavelet Multi-Resolution Analysis

The multi-resolution analysis (MRA) of wavelet is a significant property in the multilevel approximation of engineering problems [44]. The concept of MRA for square-integrable signals in the context of wavelet analysis was elaborated upon by Mallat [45]. The MRA can decompose a signal into components spanned by the scaling and wavelet basis functions at different resolutions. Any finite energy function f(t) can be expressed by:

(1) $$f\left(t\right)=\sum_{k=-\infty }^{\infty }a\left(j_{o},k\right)\emptyset_{j_{o},k}\left(t\right)+\sum_{j\;=\;j_{o}}^{\infty }\sum_{k=-\infty }^{\infty }d\left(j,k\right)\psi_{j,k}\left(t\right)$$

where $\emptyset_{j_{o},k}\left(t\right)=\;2^{-j_{0}/2}\emptyset \;\left(2^{-j_{o}}t-k\right)$ and $\psi_{j,k}\left(t\right)=\;2^{-j/2}\psi \;\left(2^{-j}t-k\right)$ are the scaled and translated version of the scaling function ∅(t) and mother wavelet ψ (t), respectively; $a\left(j_{o},k\right)$ is the $k^{th}$ approximation at the scale index $j_{o}$; and d(i,k) is the $k^{th}$ detail coefficient at scale indexj. In Equation (1), the first summation gives a low resolution or coarse approximation of f(t) at the scale index$j_{o}$. In the second summation, a higher or finer resolution function that includes more detail of f(t) is added for each j.

Generally, the MRA proposes that the scaling function has a significant role in the piecewise approximation of the continuous function f(t) and depends on the scaling index. Note that the MRA is not unique and relies on the selection of the mother wavelet function. The selection of the mother wavelet and scaling function is application-dependent; therefore, no specific selection of the mother wavelet and scaling function can be employed for all applications with the desired results. In this study, Daubechies wavelets from the orthogonal wavelets family are employed, since they have been widely implemented in vibration signals. Also, the order of the mother wavelet function is the main issue in the wavelet analysis, which is established by trial and error based on the intrinsic properties of the data [29,42,46,47,48,49]. The trial and error process resulted in the selection of DB15 among the Daubechies wavelets in the present research.

## 3. Wavelet Packet

Wavelet analysis is MRA in the time and frequency domain of a non-stationary signal. It can be regarded as an extension of the traditional Fourier transform with a modifiable window size and location [50]. WPT could be considered to be an extension of the WT, which provides a complete decomposition of both the approximation and detailed coefficients at each level. The idea of separating the signal into packets is to obtain an adaptive partitioning of the time frequency plane depending on the particular signal. Detailed information can be found in the textbook by Mallat [51]. The wavelet packet function can be defined as:

(2) $$\psi_{j,k}^{i}(t)=2^{-j/2}\psi^{i}(2^{-j}t-k)\;i=0,1,2,\ldots ,2^{j}-1$$

where a wavelet packet $\psi_{\text{j},\text{k}}^{\text{i}}\left(\text{t}\right)$ is a function of three indices with integers *i*, *j*, and *k*, denoting the modulation, the scale, and the translation parameter, respectively. Moreover, $\psi^{0}$(*t*) = ∅(t) for *i* = 0 and $\psi^{1}$(*t*) = $\psi^{\;}$(*t*) for *i* = 1. The wavelet ∅(t) is called the scaling function and $\psi^{\;}$(*t*) is called the mother wavelet function. The wavelets $\psi^{i}$ for *i* > 1 are obtained from the scaling function and the mother wavelet function as:

(3) $$\psi^{2i}=\sqrt{2}\sum_{k}h(k)\psi^{i}(2t-k)$$

(4) $$\psi^{2i+1}=\sqrt{2}\sum_{k}g(k)\psi^{i}(2t-k)$$

where g(k) and h(k) are quadrature mirror filters associated with the mother wavelet function and the scaling function. In this paper, measuring the dynamic structural response is decomposed into wavelet component functions. $2^{j}$ WPD components can be derived when the level of decomposition is *j*. The original signal can be represented as a summation of WPD components as:

(5) $$f(t)=\sum_{i=1}^{2^{j}}f_{j}^{i}(t)$$

where t is time lag; $f_{j}^{i}\left(t\right)$ is the WPD component signal that can be represented by a linear combination of wavelet packet functions as follows:

(6) $$f_{j}^{i}(t)=\sum_{k=-\infty }^{\infty }C_{j,k}^{i}\psi_{j,k}^{i}(t)$$

where $C_{j,k}^{i}$ are wavelet packet coefficients and can be calculated from:

(7) $$C_{j,k}^{i}=\int_{-\infty }^{\infty }f(t)\psi_{j,k}^{i}(t)dt$$

WPT offers good time resolution in the high-frequency band of a signal and good frequency resolution in the low-frequency band of the signal.

## 4. Identification of Time-Variant System in Terms of the Scaling Function

The force-vibration differential equations of motion of a linear time-variant system can be expressed as:

(8) $$M\ddot{x}(t)+C\dot{x}(t)+Kx(t)=f(t)$$

where M, C, and K are mass, damping, and stiffness matrices, respectively; $\ddot{x}\left(t\right)$, $\dot{x}\left(t\right)$, x(t), and f(t) are acceleration, velocity, displacement, and external force vectors, respectively.

In the Daubechies wavelet system with a given scaling function ∅(t) and corresponding wavelet function ψ(t), the acceleration ${\ddot{x}}_{h}\left(t\right)$ and the excitation $f_{h}\left(t\right)$ with the n data point can be demonstrated in terms of the scaling functions $\emptyset_{j,k}\left(t\right)$ and corresponding coefficients $a_{j,k}\left(t\right)$ and $p_{j,k}\left(t\right)$ at the $h^{th}$ degree-of-freedom, as follows:

(9) $${\ddot{x}}_{h}(t)=\sum_{k=0}^{n}a_{h}(j,k)\emptyset_{j,k}(t)$$

(10) $$f_{h}(t)=\sum_{k=0}^{n}p_{h}(j,k)\emptyset_{j,k}(t)$$

where j indicates the scale and ${\dot{x}}_{h}\left(t\right)$ and $x_{h}\left(t\right)$ can be derived using functional integration of the ${\ddot{x}}_{h}\left(t\right)$ acceleration data:

(11) $${\dot{x}}_{h}(t)=\sum_{k=0}^{n}a_{h}(j,k)\int_{-\infty }^{t}\emptyset_{j,k}(\tau )d\tau$$

(12) $$x_{h}\left(t\right)=\overset{n}{\underset{k=0}{\sum }}a_{h}\left(j,k\right)\int_{-\infty }^{t}\left[\int_{-\infty }^{\tau_{1}}\emptyset_{j,k}\left(\tau \right)d\tau \right]d\tau_{1}$$

${\dot{x}}_{h}\left(t\right)$ and $x_{h}\left(t\right)$ can be indicated in the form of ${\ddot{x}}_{h}\left(t\right)$ by multiplying $\emptyset_{j,k}\left(t\right)$ in Equations (11) and (17) and taking the integral from −∞ to +∞, respectively, as follows:

(13) $${\dot{x}}_{h}(t)=\sum_{l=0}^{n}[\sum_{k=0}^{n}a_{h}(j,k)\int_{-\infty }^{+\infty }[\int_{-\infty }^{t}\emptyset_{j,k}(\tau )d\tau ]\emptyset_{j,l}(t)dt]\emptyset_{j,l}(t)=\sum_{l=0}^{n}v_{h}(j,l)\emptyset_{j,l}(t)$$

(14) $$x_{h}(t)=\sum_{l=0}^{n}[\sum_{k=0}^{n}a_{h}(j,k)\int_{-\infty }^{+\infty }[\int_{-\infty }^{t}\left[\int_{-\infty }^{\tau_{1}}\emptyset_{j,k}(\tau )d\tau \right]d\tau_{1}]\emptyset_{j,l}(t)dt]]\emptyset_{j,l}(t)=\sum_{l=0}^{n}d_{h}(j,l)\emptyset_{j,l}(t)$$

The equation of motion in terms of discrete wavelet transform can be expressed as:

(15) $$M\sum_{k=0}^{n}a_{n}(j,k)\emptyset_{j,k}(t)+C\sum_{l=0}^{n}\sum_{k=0}^{n}a_{h}(j,k)\Gamma_{j}^{l-k}\emptyset_{j,l}(t)+K\sum_{l=0}^{n}\sum_{k=0}^{n}a_{h}(j,k)\Omega_{j}^{l-k}\emptyset_{j,l}(t)=\sum_{k=0}^{n}p_{h}(j,k)\emptyset_{j,k}(t)$$

where $\Gamma_{j}^{l-k}$ and $\Omega_{j}^{l-k}$ are the connection coefficients with respect to the certain wavelet at level j.

## 5. Connection Coefficient

From Equations (13) and (14), the unknown function $v_{h}\left(j,l\right)$ and $d_{h}\left(j,l\right)$ can be obtained from the first and second integration of the given scaling function $\;\emptyset_{j,l}\left(t\right)$. By definition, the connection coefficient is integral, with the integrand being the product of the wavelet function or scaling functions and their derivatives and integrals. The connection coefficients are defined as:

(16) $$\Gamma_{j}^{l-k}=\int_{-\infty }^{+\infty }\;\left[\int_{-\infty }^{t}\emptyset_{j,k}\left(\tau \right)d\tau \right]\emptyset_{j,l}\left(t\right)dt=\int_{-\infty }^{+\infty }\;\left[\int_{-\infty }^{t}\emptyset_{j,0}\left(\tau \right)d\tau \right]\emptyset_{j,l-k}\left(t\right)dt$$

(17) $$\Omega_{j}^{l-k}=\int_{-\infty }^{+\infty }\;\{\int_{-\infty }^{t}\left[\int_{-\infty }^{\tau_{1}}\emptyset_{j,k}\left(\tau \right)d\tau \right]d\tau_{1}\}\;\emptyset_{j,l}\left(t\right)dt=\int_{-\infty }^{+\infty }\;\{\int_{-\infty }^{t}\left[\int_{-\infty }^{\tau_{1}}\emptyset_{j,0}\left(\tau \right)d\tau \right]d\tau_{1}\}\;\emptyset_{j,l-k}\left(t\right)dt$$

With j = 0 one gets the coefficients $\Gamma_{0}^{q}$ and $\Omega_{0}^{q}$, which are called the fundamental connection coefficients for the first and second integration with regard to the l−k, respectively. For the purpose of simplicity, supposing that l−k is equal to q in Equations (16) and (17), that is:

(18) $$\Gamma_{0}^{q}=\int_{-\infty }^{+\infty }\;\left[\int_{-\infty }^{t}\emptyset_{0,0}\left(\tau \right)d\tau \right]\emptyset_{0,q}\left(t\right)dt$$

(19) $$\Omega_{0}^{q}=\int_{-\infty }^{+\infty }\;\{\int_{-\infty }^{t}\left[\int_{-\infty }^{\tau_{1}}\emptyset_{0,0}\left(\tau \right)d\tau \right]d\tau_{1}\}\;\emptyset_{0,q}\left(t\right)dt$$

From Equations (18) and (19), it can be seen that $\Gamma_{0}^{q}$ and $\Omega_{0}^{q}$ are functions of and that $\emptyset_{0,q}\left(t\right)$ is able to shift over time with the support of q, while the supports $\int_{-\infty }^{t}\emptyset_{0,0}\left(\tau \right)d\tau$ and $\int_{-\infty }^{t}\left[\int_{-\infty }^{\tau_{1}}\emptyset_{0,0}\left(\tau \right)d\tau \right]d\tau_{1}$ are fixed over time. The relations of $\Gamma_{0}^{q}$ and $\Omega_{0}^{q}$ depend on q in terms of the DB scaling function ∅(t), and can be considered in the following four conditions [8]:

(20) $$\Gamma_{0}^{q}=\{\begin{array}{r} \begin{array}{cc} 0\;q\leq -(L-1) & \end{array}\\ \begin{array}{cc} \Gamma_{0}^{q}-(L-1)<q<0 & \end{array}\\ 1-\Gamma_{0}^{-q}\;0\leq q<L-1\\ \begin{array}{cc} 1\;q\geq L-1 & \end{array} \end{array}\Omega_{0}^{q}=\{\begin{array}{r} \begin{array}{cc} 0\;q\leq -(L-1) & \end{array}\\ \begin{array}{cc} \Omega_{0}^{q}-(L-1)<q<0 & \end{array}\\ 1-\Omega_{0}^{-q}\;0\leq q<L-1\\ \begin{array}{cc} 1\;q\geq L-1 & \end{array} \end{array}$$

To determine the values of $\Gamma_{0}^{q}$ and $\Omega_{0}^{q}$ by applying the properties of Daubechies wavelets, *i.e.*, two-scale relations for the scaling function ∅(t) in Equations (18) and (19), they can be expressed as:

(21) $$\Gamma_{0}^{q}=\left(\frac{1}{4}\right)\overset{L-1}{\underset{j=0}{\sum }}\;\overset{L-1}{\underset{k=0}{\sum }}c\left(j\right)c\left(k\right)\Gamma_{0}^{-j+2q+k}$$

(22) $$\Omega_{0}^{q}=\left(\frac{1}{8}\right)\overset{L-1}{\underset{j=0}{\sum }}\;\overset{L-1}{\underset{k=0}{\sum }}c\left(j\right)c\left(k\right)\Omega_{0}^{-j+2q+k}$$

## 6. Damage Detection Approach

**Figure 1:**
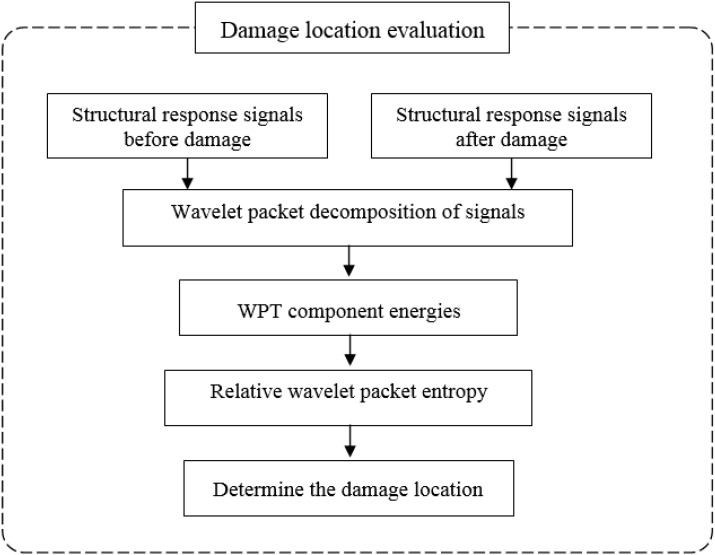
Diagram of damage location detection.

**Figure 2:**
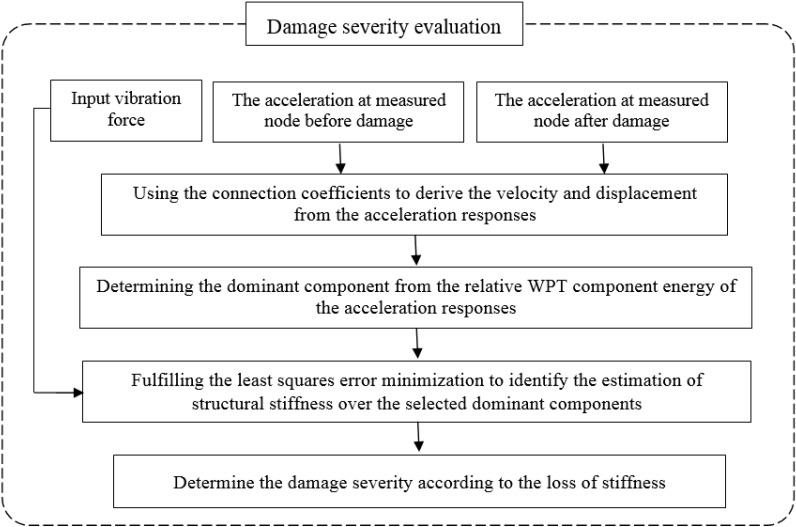
Diagram of damage severity evaluation.

The identification of vibration-based structural damage aims to compare the structural parameters obtained from measured vibration signals between the undamaged and damaged state. When structural damage occurs, a corresponding change is produced according to the damage features that evolve from the structural response signals before and after the damage. The key issue in structural damage identification is the means by which change is identified and quantified. Therefore, this study deals with the development of a hybrid approach using RWPE and system identification in the wavelet domain to optimally determine the location and severity of the damage in beam structures under flexural vibration. This approach contains two steps, *i.e.*, the first is to detect the damage locations and the second is to determine the severity of the damage in terms of the loss of stiffness. Figure 1 and Figure 2 illustrate the procedures for locating the damage and evaluating the severity, respectively.

### 6.1. Damage Location Detection

To examine the structural health condition, it is essential to achieve an index that is sensitive to structural damage. To obtain this new index, the following steps need to be pursued:

Step1: The measured vibration signals are decomposed by WPT into component signals in all frequency bands.

Step2: The WPT component energy is calculated.

The WPT component energy is an appropriate tool to identify and characterize a particular phenomenon of signal in the time-frequency domain. Sun and Chang [48] conducted a comparative study on the sensitivity of four damage indices based on the variations in frequency, mode shape, flexibility, and wavelet packet energy and deduced that the wavelet packet energy-based index has high potential for identifying the location of the damage. The sensitivity of the WPT component energy with regard to a local change in the system parameters was derived by Law *et al.* [52]. Ren *et al.* [53] studied the application of the wavelet packet energy variation-based damage detection method in bridge shear connector monitoring.

The wavelet packet energy $E_{f}$ of a signal can be defined as:

(23) $$E_{f}=\int_{-\infty }^{\infty }f^{2}\left(t\right)dt=\overset{2^{j}}{\underset{m_{1}=1}{\sum }}\;\overset{2^{j}}{\underset{m_{2}=1}{\sum }}\;\int_{-\infty }^{\infty }f_{j}^{m_{1}}\left(t\right)f_{j}^{m_{2}}\left(t\right)dt$$

where $f_{j}^{m_{1}}$ and $f_{j}^{m_{2}}$ stand for the decomposed wavelet components. The total signal energy can be expressed as the summation of the energies of the WPT components when the mother wavelet is orthogonal:

(24) $$E_{f}=\overset{2^{j}}{\underset{i}{\sum }}E_{f_{j}^{i}}=\overset{2^{j}}{\underset{i=1}{\sum }}\;\int_{-\infty }^{\infty }f_{j}^{i}{\left(t\right)}^{2}dt$$

Then, the relative energy of each packet’s components can be written as:

(25) $$p_{ij}=\frac{E_{f_{j}^{i}}}{E_{f}}$$

The $p_{ij}$values correspond to a relative energy of a particular coefficient $E_{f_{j}^{i}}$ to the total energy. The $p_{ij}$ value acts like a probability distribution of the energy. Therefore, the $p_{ij}$ values sum to one.

Step 3: The RWPE is calculated.

The Shannon entropy represents the amount of information that is also often used as a measure of the extent of the signal energy concentration in the time-frequency domain. Ren and Sun [42] applied the theory of wavelet entropy to the structural damage detection problems. The wavelet entropy spectra represent the level of order/disorder of vibration signals [43].

The damage detection problem can be formulated through the changes in the wavelet packet entropy before and after the occurrence of damage. To identify the change in vibration signals of a structure, the RWPE is defined as:

(26) $$S_{RWPE}^{k}\left(p^{k}\text{|}q^{k}\right)=\overset{\;}{\underset{j}{\sum }}\;\overset{\;}{\underset{i}{\sum }}\left|p_{ij}^{k}ln\left(\frac{p_{ij}^{k}}{q_{ij}^{k}}\right)\right|\;k=x,\;y,z.$$

It should be noted that accelerations measured in the same direction should be used in the computation of RWPE. Since the damage at a particular position affects the vibration signals in every direction, the damage index based on the RWPE is defined as:

(27) $$DI_{RWPE}=\overset{x,y,z\;}{\underset{k=1}{\sum }}S_{RWPE}^{k}\left(p^{k}\text{|}q^{k}\right)$$

However, when the structure is damaged, the values of $p_{ij\;}$ and $q_{ij}$ become different, and, consequently, the RWPE value increases. The capability of RWPE-based structural damage identification to extract the irregular information of a signal corresponding to damage is enhanced by its WPT component energy. The RWPE has significant usefulness at high frequency where the information of a high frequency level is important.

### 6.2. Damage Severity Evaluation

To obtain the damage severity in terms of the loss of stiffness in beam structures, the inverse solution of the equations of motion of a structural system in the wavelet domain is employed. As shown in Figure 2, the following steps are proposed:

Step 1: Velocity and displacement are obtained from the measured acceleration response by using the connection coefficients through Equations (21) and (22).

Step 2: The components’ energy plays a key role in determining the importance and priority of the components. Hence, to reduce the size of data without losing any important information from the signal, the dominant components are defined from the distribution of the relative energy of the WPT component at a certain level of decomposition. Consequently, the equations of motion in the time domain are converted to a form of the equation of motion in terms of WPT as:

(28) $$\text{M}U_{i,j}^{\ddot{x}}+\text{C}U_{i,j}^{\dot{X}}+\text{K}U_{i,j}^{X}=U_{i,j}^{F}$$

where $U_{i,\;j}^{Y}\;$ is the i^th^ decomposed signal at the decomposition level j of the signal Y, *i.e.*, acceleration, velocity, and displacement, in terms of WPT.

Step 3: Implement the least squares error minimization over the dominant components, which were selected in Step 2, to determine the structural stiffness with respect to the sequential number of dominant components.

(29) $$\|\text{M}U_{i,j}^{\ddot{x}}+\text{C}U_{i,j}^{\dot{X}}+\text{K}U_{i,j}^{X}-U_{i,j}^{F}\|\to \text{min}$$

Then, the severity of damage in the sense of loss of stiffness is analyzed by comparing the undamaged and damage stiffness of the beam structure.

## 7. Numerical Simulation

this section, an example of a damaged beam is given to test the proposed two-step method. For this purpose, the time history acceleration responses of the beam are computed by the finite element analysis package (ABAQUS) using a time increment of 4.8828 × 10^−4^ s, which corresponds to a sampling rate of 2048 Hz.

**Figure 3:**
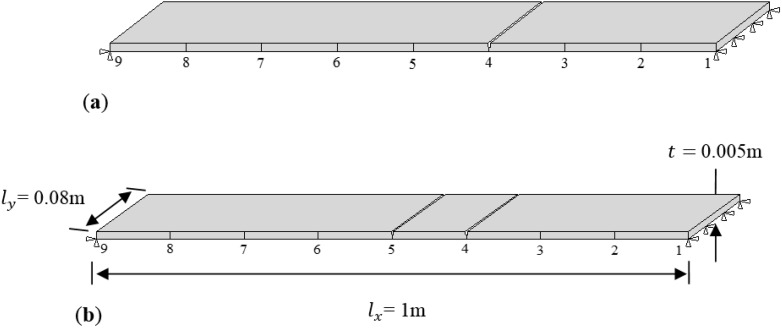
Dimensions and damage locations of plates: (**a**) Case 1; (**b**) Case2.

Consider a fixed supported beam with length $l_{x}\;$ = 1 m, width $l_{y}\;$ = 0.08 m, andhickness $t{=}^{\;}$0.005 m. The node acceleration responses of the beam under loading are obtained from nine locations on the beam, as shown in Figure 3. The force-time history is applied at location 5 on the beam. An undamaged case and two different damage cases with varying locations are investigated, as shown in Figure 3. Case 1 is the single damage scenario with damage located at point 4 while Case 2 has two points of damage, at locations 4 and 5.

### 7.1. Identification of Damage Locations

To validate the proposed method in step one, the simulated beams with predefined damage cases are considered in this study. The RWPE of a vibration signal changes as a result of structural damage and hence structural damage can be confirmed according to the change rule of RWPE.

**Figure 4:**
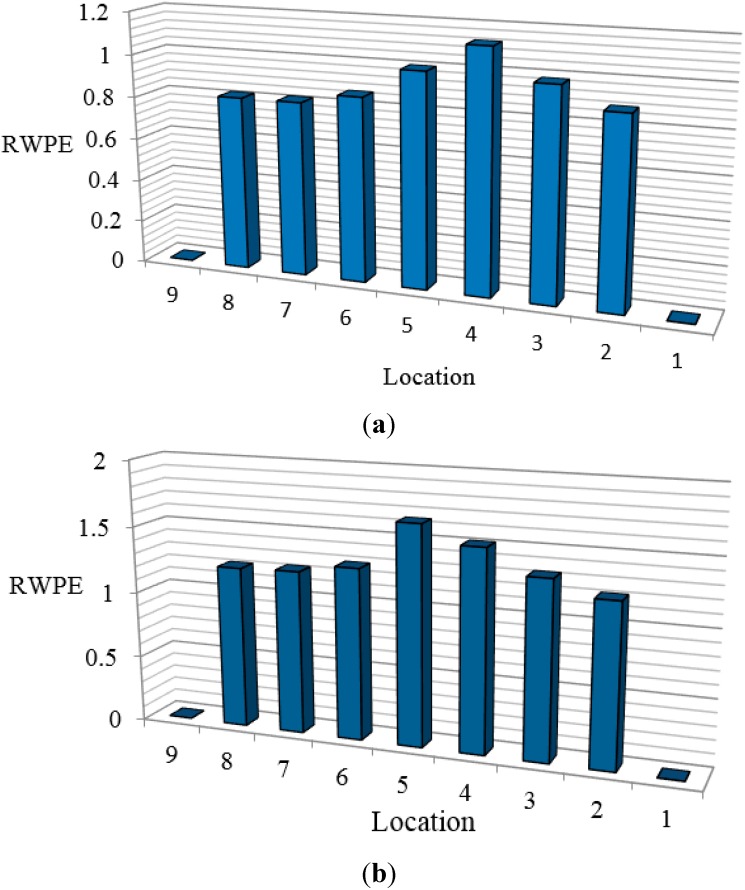
Histograms of RWPE: (**a**) Case 1; (**b**) Case 2.

For the measured data obtained from the vibration tests, the decomposition of the wavelet packet results in 128 components by setting the decomposition level to 7. Subsequently, the RWPE at every location is calculated based on Equation (29) and shown in Figure 4 for the single and multiple damage cases, respectively. According to this figure, the value and distribution of RWPEs change considerably after damage occurrence.

From these results, in damage Case 1 for the single damage scenario, it is indicated that the damage location is distinguishable in these histograms, with RWPE reaching a peak value at point4, which is the exact damage location. In addition, in Case 2, when the structure is subjected to multiple damages, by comparing the values of RWPE in Figure 4b, it can be seen that the damage locations 5 and 4 clearly stand out in the histograms. Moreover, the value of RWPE is influenced by the crack locations as well as the distance from the supports. The corresponding value of RWPE of the damage located at the middle of the beam is expected to be larger than that closer to the support, which could be due to the singularity around the support.

The results demonstrate that the locations of damage can be successfully determined from the measured time history acceleration responses through the variation of RWPE. The type of mother wavelet function plays a key role in reducing the false positives adjacent to the damage locations. This is mostly because the correlation between the mother wavelet functions and the signal is calculated as a wavelet coefficient.

**Figure 5:**
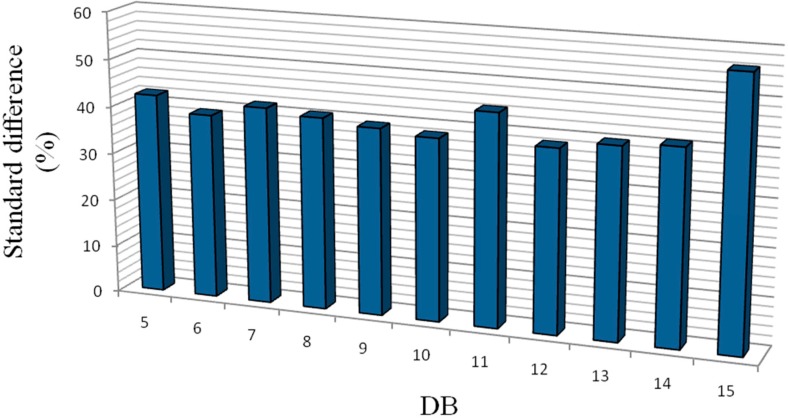
Damage identification results using different wavelet function.

In order to indicate the influence of changing the wavelet function on the accuracy of identifying the damage location, various wavelet functions DB5 to DB15 are applied for Case 1. The standard difference percentage of RWPE ([(RWPE^max^ − RWPE^ave^)/RWPE^ave^] × 100) for damage Case 1 at the specific depth of damage is calculated. The standard difference percentage is obtained in decomposition level 7, as illustrated in Figure 5. A comparison of the histograms for all considered DBs indicates that wavelet function DB15 is the suitable wavelet function order.

### 7.2. Evaluation of Damage Severity

The performance of the algorithm presented in Section 6.2 is illustrated in this section. The proposed method uses the time-history acceleration of the output response. The mass parameters are assumed to be known, since in the numerical simulation, the true structural parameters of the original model are known. The proposed method can be evaluated by studying the accuracy in terms of the percentage loss of stiffness between the identified structural parameters and the true primary values.

**Figure 6:**
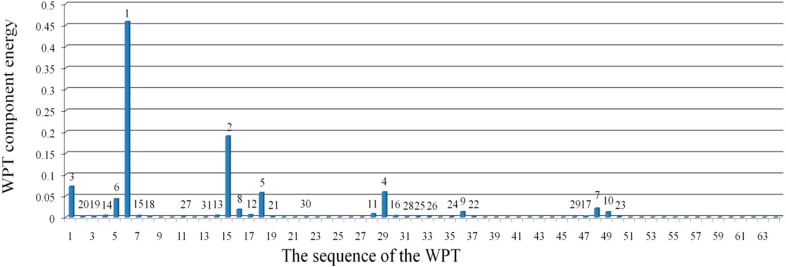
The relative energy distribution of the acceleration response for the undamaged case.

To investigate the proposed algorithm based on the WPT, Figure 6 depicts the distribution of the relative energy of the acceleration response for the undamaged case at decomposition level 6. These components provide information about the relative energy associated with the various frequency bands existing in the response signals. Table 1 shows the frequency bands of the corresponding WPT components.

**Table 1:** Frequency bands of the WPT components at level 6 and modal properties of the undamaged case.

The sequence of WPT	1	2	3	…	6	…	15	…	29	…	48	…	64
**Frequency bands of WPT components (Hz)**	0–16	16–32	32–48	…	80–96	…	224–240	…	448–464	…	752–760	…	1008–1024
**Natural frequencies (Hz)**	9.29				83.65		232.48		456.03		754.64		
**Damping ratios**	0.01				0.01		0.025		0.049		0.081		

The Rayleigh model is employed to calculate the damping matrix:

$$C=a_{1}K+a_{2}M$$

where a_1_ and a_2_ are the mass-proportional and the stiffness-proportional constants, respectively. The two constants of the Rayleigh damping matrix have been chosen so as to have a 1% damping ratio for the frequencies of the first two modes. The modal properties of the model are demonstrated in Table 1.

From the distribution of the component energies in Figure 6, it can be observed that the sixth WPT coefficient with the frequency band of 80–96 Hz, which includes the natural frequency 83.65 Hz, has the maximum WPT component energy among the others. Consequently, the WPT coefficients that contain the principal frequencies of the signal provide the greatest energy associated with the signal.

In addition, based on the distribution of relative energy at the given decomposition level of a signal, the dominant components are determined as:

$$D_{c}=\left\{s_{1},s_{2},\ldots ,s_{c}\right\}$$

where $s_{c}$ indicates the sequence number of the WPT coefficient having the $c^{th}$ highest relative energy, as shown in Figure 6. At a certain level of decomposition, a dominant component can be a subset of the D = {1, 2, …, 2^j^} of WPT coefficients. Technically, when the input and output data are too large to be processed and are suspected to be redundant, the data are transformed into a reduced representation set of features. If the extracted features are carefully chosen, the features set will contain the relevant information from the input and output data, and make it possible to conduct the desired task using this reduced representation instead of the full size of input and output data.

Therefore, the purpose of defining the dominant components is to present the reduced representation component in order to perform the least squares error minimization over the dominant components. In structural dynamics, the responses of a linear model consist of a linear combination of modal responses and the coefficients of modal vectors. Sometimes, in the presence of a node in the modal vectors, a response does not have the contribution from the corresponding modal response, which, instead, can be quite important. Therefore, in defining the dominant components, it is important to consider multiple responses rather than one specific response. Based on the fact that the dominant components can be obtained by the relative energy distribution over the wavelet coefficients of the system's acceleration responses, the equations of motion for the system at the given level can be transformed into those in terms of the dominant components, as shown in Equation (28).

**Figure 7:**
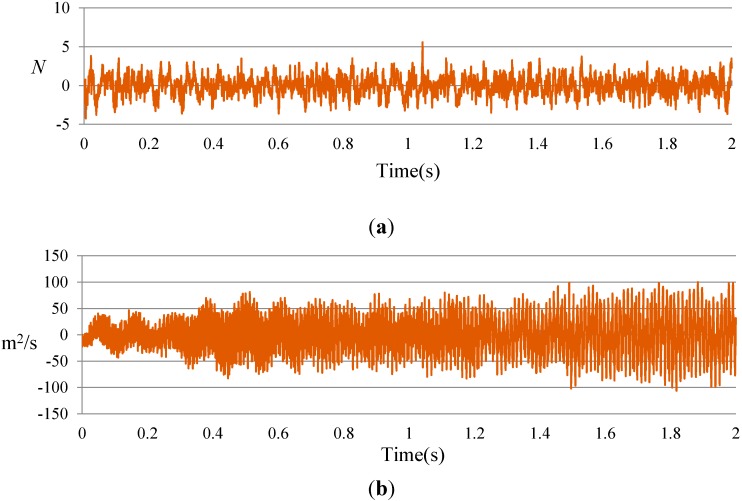

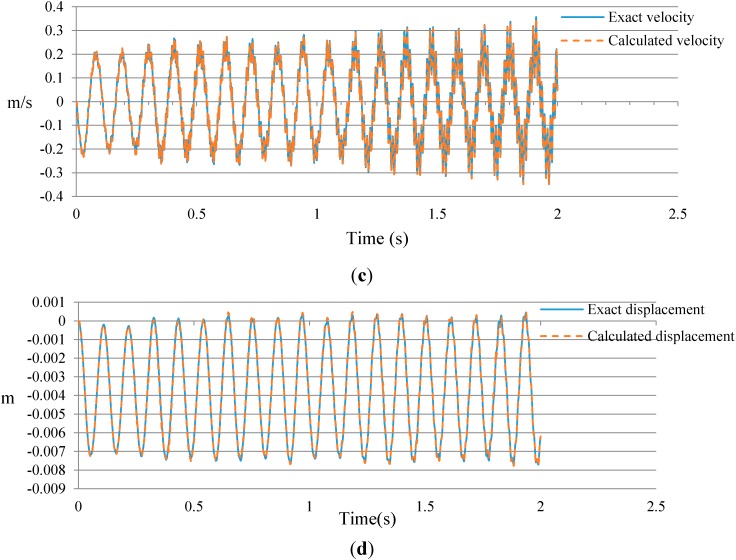
Input and response data at location 5 for undamaged case: (**a**) input force; (**b**) exact acceleration responses; (**c**) comparison of the exact velocity to the calculated velocity; (**d**) comparison of the exact displacement to the calculated displacement.

To identify the stiffness based on the inverse solution of equations of motion of a structural system in the wavelet domain, the input force and response data containing acceleration, velocity, and displacement are required. For this purpose, by using the connection coefficients of the scaling function, as expressed in Equations (21) and (22), the velocity and displacement are obtained from the measured acceleration responses at location 5 in terms of the DB15. In order to validate the accuracy of the purposed solution method, these velocities and displacements are compared with those obtained from the explicit dynamic analysis conducted using ABAQUS software, as depicted in Figure 7.

### 7.3. Discussion on the Considered Cases

**Figure 8:**
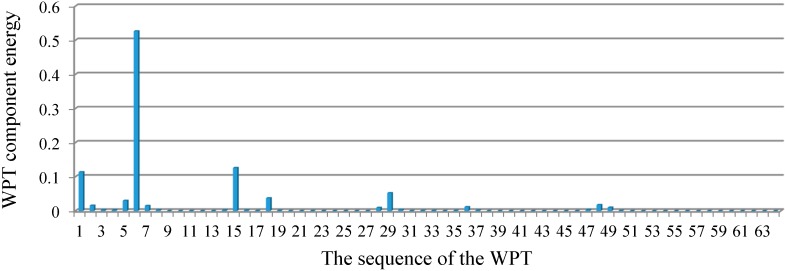
The relative energy distribution of the acceleration response for Case 1.

**Figure 9:**
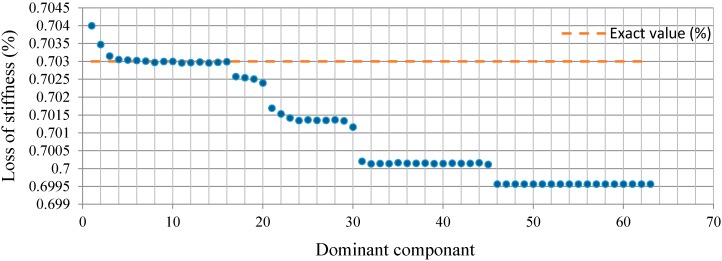
Loss of stiffness identification in Case 1.

From the above analysis, for Case1 with the single damage scenario, the distribution of relative energy of acceleration responses at decomposition level 6 is demonstrated in Figure 8. The best estimation for the structural stiffness at each dominant component, e.g., D_1_ = {6}, D_2_ = {6, 15}, …, D_10_ = {6, 15, 1, 29, 18, 5, 48, 16, …, 36, 49}, is obtained by implementing the least squares procedure iteratively over the dominant components. Therefore, the results converge to the exact values. To evaluate the damage severity in terms of the loss of stiffness, as depicted in Figure 9, it can be found that the estimated percentage values converge to the exact ones, presented by a red-colored dashed line at each dominant component beginning from D_4_. In addition, the obtained value at dominant component D_16_ in terms of the percentage loss of stiffness is the best and most accurate among all the dominant components. Moreover, with the increment in dominant components to D_17_ or higher orders, the estimated values degrade, as shown in Figure 9. This can be due to the numerical integration error in the derived displacement responses. In addition, the percentages for the loss of stiffness in Case1 between the damaged and undamaged structure, in terms of dominant components D_15_, D_16,_ and D_17_, are 0.702977%, 0.702992%, and 0.702577%, respectively. Generally, the accumulation of error in a numerical integration occurs for two reasons. The first source is the local error, which appears at each step of the integration, while the second source is caused by the cumulative effect of previous errors, which stems from many integrations. Since the proposed method depends on the numerical integration procedure, these types of error are unavoidable.

**Figure 10:**
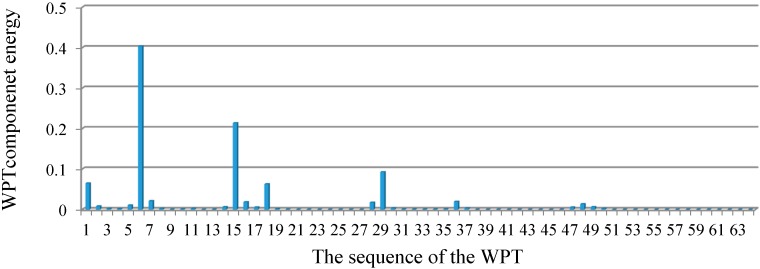
The relative energy distribution of acceleration response for Case 2.

**Figure 11:**
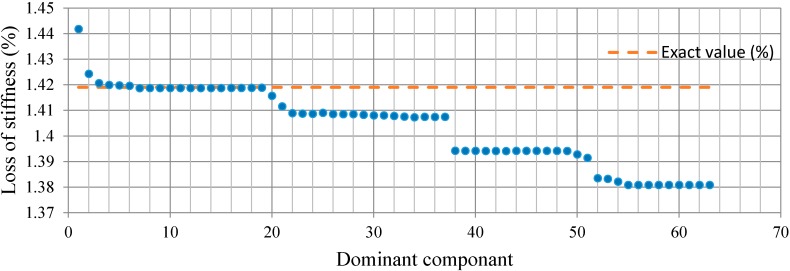
Loss of stiffness identification in Case 2.

For Case 2, with the two-damage scenario, the relative energy distribution of the acceleration response at decomposition level 6 is depicted in Figure 10. To optimize the value of each dominant component, the least squares error minimization was performed. Figure 11 shows the convergence of the percentage loss of stiffness for each component together with the exact values. According to Figure 11, the onset of convergence takes place around D_4_ and the most accurate result is associated with D_19,_ with the appropriate value of 1.4188%. From dominant component D_20_, with the value of 1.4156% onward, the convergence keeps decreasing.

The above results indicate that the proposed algorithm is effective in evaluating the damage severity in terms of the loss of stiffness. In addition, the results demonstrate that the identification of stiffness parameters can be achieved with less data.

## 8. Experimental Verification

**Figure 12:**
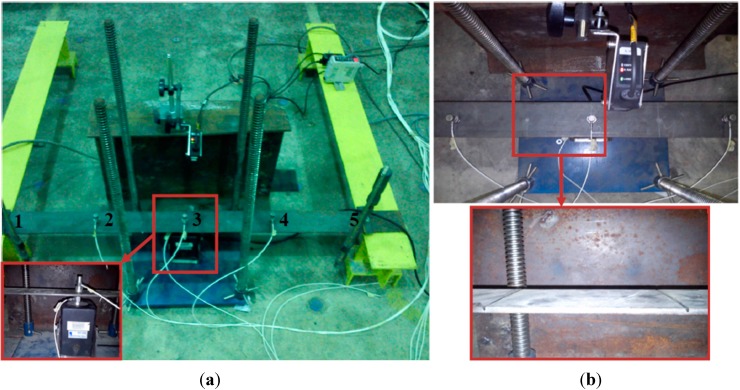
(**a**) Experimental setup and data acquisition system; (**b**) damage locations of tested beam.

To verify the effectiveness of the proposed method, an experimental study was carried out on a steel beam with fixed supports, as shown in Figure 12. The beam was one meter in length from support to support, 0.08 m width, and 0.005 m thick. The mass was considered to be known and equal to 3.207 kg. Local structural damage was simulated by cutting slots in the beam at the selected locations, as described in Table 2.

The model was divided into four intervals along the X axis; each interval was 0.25 m long, which produced three nodes in the middle and one node at each support. The acceleration of each node was measured in the Y axis using K-Shear Kistler accelerometers with frequency bands of 0.5 to 10 kHz and a sensitivity of 100 mV/g.

**Table 2:** Damage scenarios.

Damage Case	Damage Scenario	Damage Location	Width of Cut (mm)	Depth of Cut (mm)
Case 0	Undamaged	-	-	-
Case 1	Single	Between nodes 2 and 3	2	2
Case 2	Double	Between nodes 2 and 3, node 3	2	2

**Figure 13:**
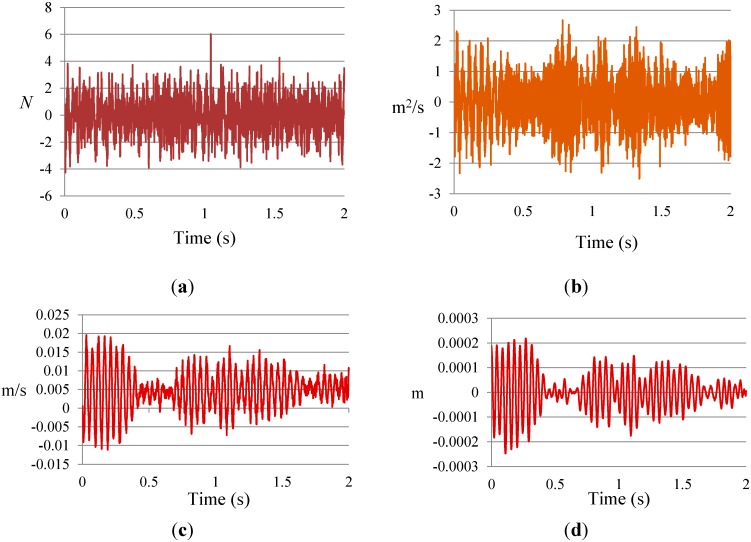
Input and structural response data at location 3 of Case 1: (**a**) force; (**b**) acceleration; (**c**) velocity; (**d**) displacement.

The beam was excited at node three, in the middle of the beam, using a Labworks ET-132 shaker which has a rated force of 22 N. The input force was measured using a PCB 208C02 force transducer with a measurement range of 449 N in both tension and compression and a frequency range of 0.001 Hz to 36 kHz. The analogue data from the sensors was converted and recorded using an OROS-OR35 data logger/analyzer. The signal analyzer, which is capable of generating all different forms of signals, including white noise, was used in this test. The sampling rate was set to 5.24 kS/s with a frequency band width of 2048 Hz. Figure 13 shows the input force and output acceleration *versus* time at location 3 of Case 0 (undamaged). Velocity and displacement were obtained from the acceleration response in terms of DB15 scaling function.

### Experimental Results

**Figure 14:**
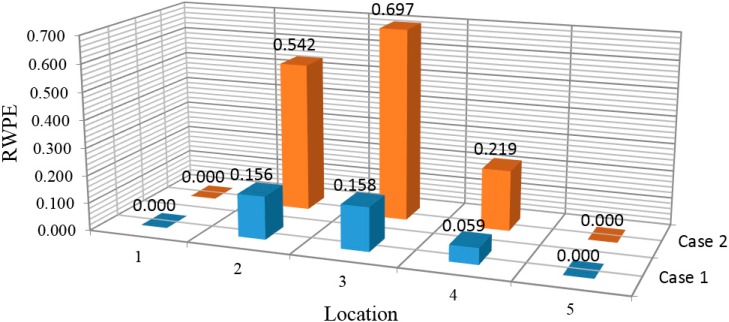
Histogram of RWPEs in two damage cases.

For the evaluation of the damage location from the measured acceleration responses, the RWPE was implemented for each damage scenario. In order to reduce the false positives caused by uncertainty due to the environmental condition and operational changes, and also to accurately identify the damage location, DB15 and decomposition level 6 were chosen by trial and error. Figure 14 shows the histogram of RWPEs in two damage cases. In Case 1, where the damage was located between two sensors, the damage location could be clearly identified with the change in values of RWPE. From the histograms, it can be observed that the magnitude of RWPE at location 3 was relatively larger than at location 2. This is possibly due to the singularity effect arising from the support. In addition, the large values of RWPE in Case 2 show the damage locations precisely. The results demonstrate that the locations of damage can be successfully determined from the measured time history acceleration responses through the variation of RWPE.

**Figure 15:**
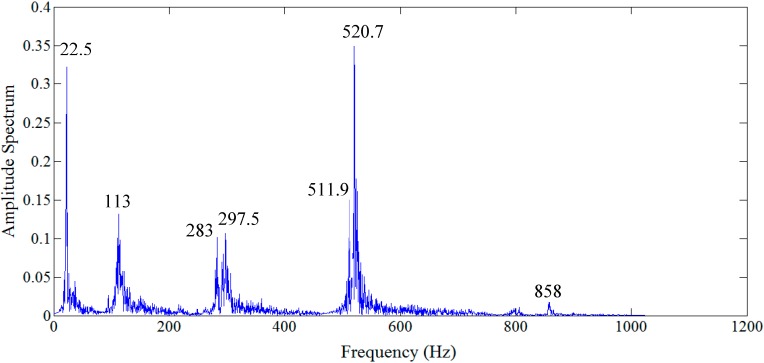
Frequency domain response of acceleration response signal at location 3.

**Figure 16:**
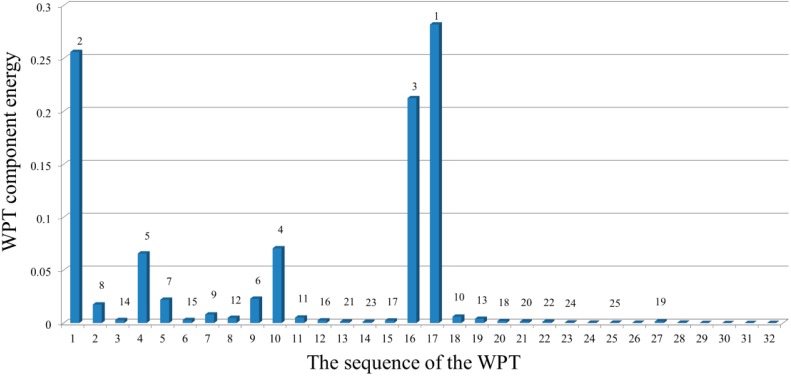
Relative energy distribution over the wavelet packet components at level 5.

After identification of the damage locations, the analysis continued to evaluate the severity of the damage. The optimization scheme was employed for the identification of damage severity in terms of the loss of stiffness. For this purpose, the relative energy distributions of the acceleration response related to the structural frequencies (Figure 15) were achieved at level 5 of decomposition, as depicted in Figure 16. Among these distributions, the WPT component energy of 17 and one, whose frequency bands are 512–544 Hz and 0–32 Hz, respectively, are larger than the others. Therefore, the dominant components are D_1_ = {17}, D_2_ = {17, 1}, …, D_5_ = {17, 1, 16, 10, 4} and so on. By applying the least squares error minimization in dominant components, the stiffness values corresponding to each dominant component can be obtained.

**Figure 17:**
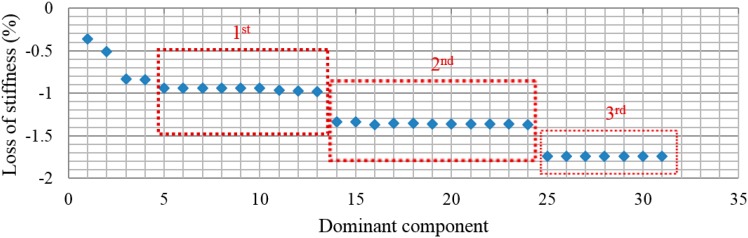
Loss of stiffness identification in each dominant component in Case 1.

With respect to the accuracy of the identification results, Figure 17 shows the percentage loss of stiffness in each dominant component for Case 1. It appears that the obtained values have converged at three regions, as illustrated in Figure 17. Based on the numerical output, the best and appropriate results can be found in the first converged region, whereas in the second and third regions, more uncertainty is included due to the inclusion of higher-order dominant components. This is because the large input and output data are considered to be redundant; therefore, it is possible to obtain accurate results by applying the dominant components instead of the full size of data. The percentage loss of stiffness in the first convergence region, in terms of each dominant component, is presented in Table 3. In the first region, the estimated values begin to converge at D_5_ and deviate from D_13_ onward.

**Figure 18:**
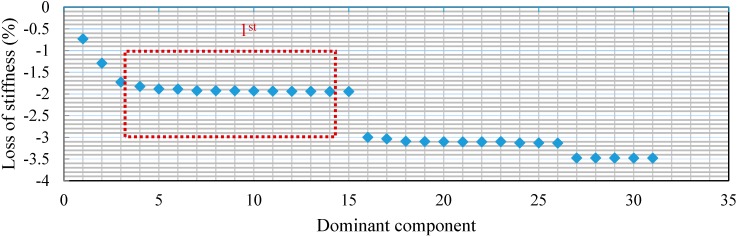
Loss of stiffness identification in each dominant component in Case 2.

In Case 2, with two damage scenarios, the percentage loss of stiffness in each dominant component is shown in Figure 18. The first convergence region with an appropriate result has been specified with respect to the accuracy of the identification parameters. The estimated results start to converge at dominant component D_5_. In addition, the obtained percentage loss of stiffness corresponding to D_15_ is found to be the best result with the highest possible precision in the indicated convergence region, as given in Table 3. Other higher-order dominant components, e.g., from D_16_ onward, are reduced.

**Table 3:** Estimated values of loss of stiffness in the first convergence region.

Dominant Component	Loss of Stiffness (%)	
	Case 1	Case 2
D_5_	−0.93827	−1.88349
D_6_	−0.93947	−1.88897
D_7_	−0.9395	−1.9238
D_8_	−0.944	−1.92455
D_9_	−0.94213	−1.9252
D_10_	−0.94491	−1.93284
D_11_	−0.96649	−1.9363
D_12_	−0.97848	−1.93921
D_13_	−0.98642	−1.94082
D_14_	−1.33572	−1.94314
D_15_	−1.34168	−1.94331

## 9. Conclusions

A two-step approach was proposed to optimally determine the location and severity of damage in the beam structure under flexural vibration. The damage locations were identified accurately in the first step by proposing a new damage index through the vibration responses based on the WPT, which were combined with the information entropy to gain the advantages of both techniques. In addition, the effects of the damage location were evaluated.

The system identification based on the wavelet multi-resolution analysis was proposed to identify the severity of damage by investigating the accuracy of the result in terms of the loss of stiffness in the model between the parameters obtained before and after the damage occurrence. The connection coefficients of the scaling function were used to obtain the velocity and displacement from the measured acceleration responses. Although this numerical integration process may inevitably introduce error to the stiffness parameters identified, by applying the dominant components based on the relative energy of acceleration responses, the best estimation of the stiffness parameter was obtained using the least squares error minimization over each dominant component. It is noteworthy that in real applications the implementation of the least squares error minimization method is not required from the first dominant component. To avoid time-consuming iterative computations for convergence and in order to obtain an accurate result with less data, the least squares error minimization can be performed across the dominant components until the first convergence region is achieved. Both the numerical simulation and experimental tests showed that the proposed method could accurately identify the locations of damage as well as the damage severity in terms of loss in stiffness.

Although the proposed damage identification methodology has shown great potential in the simulated and the laboratory-tested beam, the proper selection of wavelet functions was determined by trial and error based on the intrinsic properties of the data. However, further studies can be conducted to apply the computational intelligence methods to optimize the algorithm so as to determine the proper selection of “wavelet functions” and choosing an appropriate “resolution level”. These are practical aspects that should be studied further so that a RWPE-based structural damage identification method can be applied with confidence to real structures.
